# Simulation of the mortality after different ex ante (secondary) and ex post (tertiary) triage methods in people with disabilities and pre-existing diseases

**DOI:** 10.1007/s00101-023-01336-7

**Published:** 2023-09-21

**Authors:** Sara Garber, Jens O. Brunner, Axel R. Heller, Georg Marckmann, Christina C. Bartenschlager

**Affiliations:** 1 Working Group for Health Care Operations/Health Information Management, Faculty of Business and Economics, Faculty of Medicine, University of Augsburg, Universitätsstr. 16, 86159 Augsburg, Germany; 2 Professor of Decision Science in Healthcare, Department of Technology, Management, and Economics, Technical University of Denmark, Lyngby, Denmark; 3 Clinic for Anaesthesiology and Operative Intensive Care, Medical Faculty, University Hospital of Augsburg, University of Augsburg, Stenglinstr. 2, 86156 Augsburg, Germany; 4 Institute for Ethics, History and Theory of Medicine, Ludwig-Maximilians-Universität München, Lessingstr. 2, 80336 Munich, Germany; 5 Professor of Applied Data Science in Healthcare, Nürnberg School of Health, Klinikum Nürnberg and Ohm University of Applied Sciences Nuremberg, Keßlerplatz 12, Nuremberg, Germany

**Keywords:** Ethics, Life value equality, Age, Probability of survival, Scores, Ethik, Lebenswertgleichheit, Alter, Überlebenswahrscheinlichkeit, Scores

## Abstract

The significant increase in patients during the COVID-19 pandemic presented the healthcare system with a variety of challenges. The intensive care unit is one of the areas particularly affected in this context. Only through extensive infection control measures as well as an enormous logistical effort was it possible to treat all patients requiring intensive care in Germany even during peak phases of the pandemic, and to prevent triage even in regions with high patient pressure and simultaneously low capacities. Regarding pandemic preparedness, the German Parliament passed a law on triage that explicitly prohibits ex post (tertiary) triage. In ex post triage, patients who are already being treated are included in the triage decision and treatment capacities are allocated according to the individual likelihood of success. Legal, ethical, and social considerations for triage in pandemics can be found in the literature, but there is no quantitative assessment with respect to different patient groups in the intensive care unit. This study addressed this gap and applied a simulation-based evaluation of ex ante (primary) and ex post triage policies in consideration of survival probabilities, impairments, and pre-existing conditions. The results show that application of ex post triage based on survival probabilities leads to a reduction in mortality in the intensive care unit for all patient groups. In the scenario close to a real-world situation, considering different impaired and prediseased patient groups, a reduction in mortality of approximately 15% was already achieved by applying ex post triage on the first day. This mortality-reducing effect of ex post triage is further enhanced as the number of patients requiring intensive care increases.

**Supplementary Information:**

The online version of this paper (10.1007/s00101-023-01336-7) contains supplemental tables S1 and S2.

## Abstract (de)

Der stetige Anstieg an zu behandelnden Patienten während der COVID-19-Pandemie hat das Gesundheitssystem vor eine Vielzahl an Herausforderungen gestellt. Die Intensivstation ist einer der in diesem Zusammenhang besonders stark betroffenen Bereiche. Nur durch umfangreiche Infektionsschutzmaßnahmen sowie einen enormen logistischen Aufwand konnten in Deutschland selbst in Hochphasen der Pandemie die Behandlung aller Intensivpatienten ermöglicht und eine Triage auch in Regionen mit hohem Patientendruck bei gleichzeitig geringen Kapazitäten verhindert werden. Im Hinblick auf die Pandemievorsorge hat der Deutsche Bundestag ein Gesetz zur Triage verabschiedet, das eine Ex-post-Triage explizit untersagt. Bei einer Ex-post-Triage werden auch Patienten, die bereits auf der Intensivstation behandelt werden, in die Triage-Entscheidung einbezogen und Behandlungskapazitäten nach individueller Erfolgsaussicht verteilt. In der Literatur finden sich rechtliche, ethische und soziale Überlegungen zur Triage bei Pandemien, eine quantitative Bewertung im Hinblick auf verschiedene Patientengruppen auf der Intensivstation gibt es hingegen nicht. Der Fokus der Arbeit liegt auf dieser Forschungslücke, und es wird eine quantitative simulationsbasierte Evaluation von Ex-ante- und Ex-post-Triage-Politiken unter Berücksichtigung von Überlebenswahrscheinlichkeiten, Beeinträchtigungen und Vorerkrankungen durchgeführt. Die Ergebnisse zeigen, dass eine Anwendung von Ex-post-Triage, basierend auf Überlebenswahrscheinlichkeiten in allen Patientengruppen, zu einer Reduktion der Mortalität auf der Intensivstation führt. In dem Szenario, das der realen Situation wohl am nächsten kommt, wird eine Reduktion der Mortalität auf der Intensivstation um ca. 15 % schon bei einer einmaligen Anwendung der Ex-post-Triage erreicht. Dieser mortalitätsreduzierende Effekt ist umso größer, je mehr Patienten auf eine intensivmedizinische Behandlung warten.

## Introduction

The significant increase in patients during the coronavirus disease 2019 (COVID-19) pandemic presented the healthcare system with a variety of challenges. The intensive care unit (ICU) was one of the areas particularly affected in this context. Only through extensive infection control measures as well as an enormous logistical effort [1] was it possible to treat all patients requiring intensive care in Germany even during peak phases of the pandemic, and to prevent triage even in regions with high patient pressure and simultaneous low capacities [2]. In the context of insufficient available resources, the term “prioritization decisions when resources are scarce” [3] is often used. Although being standard in international literature, the term triage until now is replaced in Germany by the term *Sichtung* for historical reasons. As the term triage is synonymously frequently used in the German Parliament and in the media, the term is also used in this article.

Regarding pandemic preparedness, the German Parliament passed a law on triage that does not exclude ex ante (secondary) triage, but explicitly prohibits ex post (tertiary) triage. The Infection Protection Act (§ 5c IfSG para. 2, sentence 4) specifically states (translated): “Intensive care treatment capacities that are already allocated and essential for survival are excluded from the allocation decision”. In ex post triage, patients who are already being treated are included in the triage decision and treatment capacities are allocated according to the individual likelihood of success [2, 3]. This can result in a patient’s treatment in the ICU not being continued in favor of intensive care treatment for another patient with a better chance of a favourable ICU outcome. One goal of the new law was to prevent discrimination against patients with impairments, i.e., disabilities, and pre-existing conditions. In this respect, only the current and short-term probability of survival may be considered in the allocation of ICU capacities [4].

Legal, ethical, and social considerations for triage in pandemics can be found in the literature [3, 5–9] but there is no quantitative assessment with respect to different patient groups in the ICU. This study addressed this research gap and a quantitative simulation-based evaluation of the impact of different ICU control policies was conducted. Using a simulation model, the effects of different ex ante and ex post triage policies on ICU mortality were evaluated. In particular, the focus was on the effects for different patient groups, i.e., patients without impairments and pre-existing conditions and patients with impairments and pre-existing conditions. The work was based on data from the most recent literature during the COVID-19 pandemic and provides support for decision-makers in ICU planning and management during situational overload of the medical system when sufficient medical care can no longer be provided to all patients.

## Methods

**Fig. 1:**
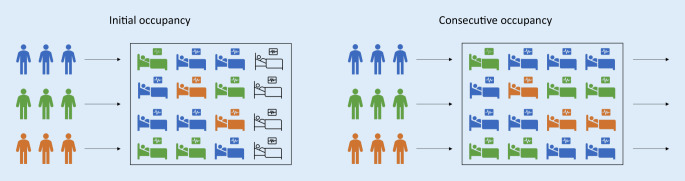
Graphical representation of the decision problem for different exemplary patient groups. *Blue* patient group 1, *green* patient group 2, *orange* patient group 3 in the intensive care unit: Initial occupancy with still free treatment capacities. These can be filled with newly arriving patients (consecutive occupancy). After that, all treatment bays are occupied.

The study simulated an intensive care unit with COVID-19 patients. A distinction was made between patients with or without additional comorbidity or disability. The number of patients to be treated exceeded the number of ICU beds, i.e., a decision on which patients should be treated was to be made (Fig. 1). Thereby, an ICU bed was defined as a fully staffed and equipped ICU treatment capacity capable of providing organ support and, if necessary, organ replacement for one patient at a time.

The decision as to which patients could be (further) treated was made randomly or based on survival probabilities, depending on the triage policy. The focus was on the effects for different groups of patients on the ICU. In the following, the underlying data set, the triage policies, i.e., control policies, as well as the simulation model and its evaluation are described.

### Dataset

This study was based on data from most recent literature during the COVID-19 pandemic. For patients without impairments and pre-existing conditions, the (normalized) values of the Robert Koch Institute (RKI) were used to generate the probabilities of death [10]. The probability of death was defined as the complement to the probability of survival. A triangular distribution was assumed, because, for this distribution the measures identified by the RKI [10] could be implemented intuitively from an application perspective^1^:

$$X\ \Updelta (0.04;0.41;0.045)$$

with^2^

$$E\left(x\right)=0.165$$

The triangular distribution, where the name verbalizes its visualization, was thereby defined by minimum, maximum, and most likely values (mode). Subsequently, (adjusted) literature-based proportionates for the (approximate) relative risk of death [11–15], i.e., odds ratio and hazard ratio, were applied to the distribution and its expected value, respectively. This resulted in the triangular distributions for generating the probabilities of death of patients with impairments and pre-existing conditions. In the simulation, two impairments, i.e., trisomy 21 and amyotrophic lateral sclerosis, and three pre-existing conditions, i.e., cardiovascular disease, hypertension, and diabetes mellitus (type 2), were considered. When considering impairments, amyotrophic lateral sclerosis was chosen, because this impairment was of special importance in the discussion against ex post triage. Trisomy 21 was chosen, because proportionates related to COVID-19 risk factors in the literature. The following values were used as odds ratios or hazard ratios: risk of ICU treatment (trisomy 21), 30-day mortality risk (amyotrophic lateral sclerosis), in-hospital mortality (cardiovascular disease), relative risk of death (hypertension), and risk of in-hospital death from COVID-19 (diabetes mellitus, type 2). For the sake of simplicity, these values were hereafter referred to as the relative risk of death. Among the impairments and pre-existing conditions considered, the proportionate for relative risk of death is highest for cardiovascular disease at 4.85, whereas this is lowest for diabetes mellitus (type 2) at 2.03. An overview of all literature-based proportionates for relative risk of death used is shown in Table 1.

**Table 1:** Overview of the data applied from COVID-19 literature for the relative risk of death

Focus	Paper	Proportionate	Applied values
Trisomy 21	Bergman et al. (2021) [11]	Odds ratio	$4.52;CI(3.13;7.36)$^a^
Cardiovascular disease	Li et al. (2020) [12]	Odds ratio	$4.85;CI(3.07;7.70)$
Hypertension	Gao et al. (2020) [13]	Hazard ratio	$2.12;CI(1.17;3.82)$
Diabetes mellitus (type 2)	Barron et al. (2020) [14]	Odds ratio	$2.03;CI(1.97;2.09)$
Amyotrophic lateral sclerosis	Galea et al. (2021) [15]	Odds ratio	$3.00;CI(1.9;4.9)$

^a^Reduction of confidence interval (CI) by 40%

**Fig. 2:**
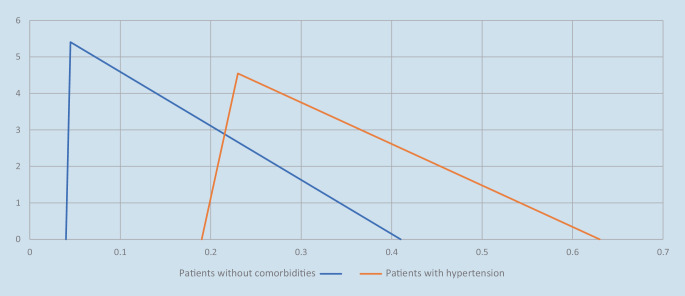
Density functions (ordinate) of the probability of death (abscissa) of patients without comorbidities and patients with hypertension

The parameters of the triangular distribution, i.e., minimum, maximum, and mode, used to generate the probability of death for patients with impairments and pre-existing diseases were set in such a way that it was possible to apply the ratio measures to the expected value. At the same time, it was ensured that impaired patients and those with pre-existing diseases could have a higher probability of survival than patients without impairments and pre-existing conditions. Figure 2 shows the density functions of the probability of death^3^ of patients without impairments or pre-existing conditions and patients with hypertension.

Using the proportionate, the triangular distribution for patients with hypertension was as follows:

$$X\ \Updelta \left(0.19;0.63;0.23\right)\ \text{with}\ E\left(X\right)=0.35$$

Thus, the proportion of overlapping of the two density functions in this case was 31.35%.

### Triage policies

To compare the effects of different control policies on ICU mortality, different combinations of ex ante and ex post triage^4^ were simulated. The probability of death (counter probability to the probability of survival) was used as the decisive criterion for the triage decision. Considering the probability of survival, which can be calculated based on medical scores and may be used as the only criterion according to the current legislation, provides a superior result compared to other criteria such as age [2]. In the simulation, initial occupancy procedures, i.e., initial random occupancy and initial ex ante triage based on survival probabilities, were combined with consecutive occupancy procedures, i.e., random consecutive occupancy and consecutive ex post triage based on survival probabilities. The combinations of the respective control policies are shown in Table 2.

**Table 2:** Overview of the control policies applied

Policy	Description
0	Initial random occupancy, random consecutive occupancy
1	Initial random occupancy, consecutive ex post triage based on survival probabilities
2	Initial ex ante triage based on survival probabilities, random consecutive occupancy
3	Initial ex ante triage based on survival probabilities, consecutive ex post triage based on survival probabilities
4	90*%* initial random occupancy / 10*%* initial ex ante triage based on survival probabilities, random consecutive occupancy (current legislation)
5	90*%* initial random occupancy / 10*%* initial ex ante triage based on survival probabilities, consecutive ex post triage based on survival probabilities

In policy 0 neither ex ante nor ex post triage based on survival probabilities was performed. The policy thus served as a benchmark for all other control policies. Policies 1 and 2 each involved only one form of triage based on survival probabilities. Policy 2 provided a lower bound on ICU mortality because initial ex ante triage based on survival probabilities to occupy the entire ICU is unlikely to ever be performed in practice. Nonetheless, policy 2 was able to demonstrate the maximum effect that could be achieved by using initial ex ante triage based on survival probabilities. A combination of ex ante and ex post triage based on survival probabilities was depicted in policy 3. In addition, 2 further policies, 4 and 5, were introduced for realistic representation. Here, an ICU already filled to 90% (random occupancy) was assumed and an initial ex ante triage based on survival probabilities was performed to occupy the remaining 10% of beds available. Based on this, either random consecutive occupancy (policy 4), which is probably closest to an approach under current legislation, or consecutive ex post triage based on survival probabilities (policy 5) was used.

### Simulation model and evaluation

For the simulation study in the statistical software R (Statistical software, The R Foundation for Statistical Computing, Vienna, Austria), six different simulations were run for each of the six control policies considered. The simulations differed in the consideration of different patient groups or their combination. Thus, one simulation was performed for each of the 5 comorbidities considered, assuming that 30% of the patients were not impaired or had pre-existing diseases and 70% of the patients suffered from the impairment or pre-existing condition considered [16] as well as a real-world simulation. In the real-world simulation, all impairments and pre-existing conditions considered could occur within the group of patients with comorbidities. This approach allowed both an individual assessment of the impact of a particular impairment or pre-existing condition and the evaluation of a realistic scenario in which different groups of patients were treated on the ICU.

In the following, the structure of the real-world simulation is presented as all comorbidities were taken into account in this simulation. The procedure of the other simulations is similar but no distinction was made within the patient group with impairments and pre-existing conditions. At the beginning of the real-world simulation, the number of simulation runs $R=10{,}000$, control policies $P=6$, time points considered $T=3$^5^, the demand at the time of initialization $d=70$, and the length of the queue considered $w=10$, available intensive care beds $B=60$ and the proportion of patients with impairments or pre-existing conditions $s=0.7$, were specified. The assumption of $B=60$ beds was based on the operable intensive care capacities at Augsburg University Hospital in a COVID-19 peak situation. In each simulation run, the initialization was followed by the generation of patients from which the initial occupancy of the ICU was determined. In the case of initial random occupancy 60 patients were randomly generated, in the case of initial ex ante triage based on survival probabilities the demand at the time of initialization was generated in terms of those patients who required intensive care treatment^6^. Each patient $i\in I$ was assigned a probability of death, based on a triangular distribution, depending on whether or what impairment or pre-existing condition *k* was present:

$$p_{i}\ \Updelta \left({a}_{i}^{k}{,}{b}_{i}^{k}{,}{c}_{i}^{k}\right)\ \forall i\in I$$

Thus, a dependency was modelled on whether the patient had a comorbidity or what comorbidity the patient had^7^. In addition, *x*_*i*_ was introduced to indicate whether a patient was impaired or had pre-existing disease:

$$x_{i}=\begin{cases} 1 & if\,\textit{patient}\,i\\&\textit{is impaired}\\&\,\textit{or prediseased}\\ 0 & else \end{cases}$$

**Fig. 3:**
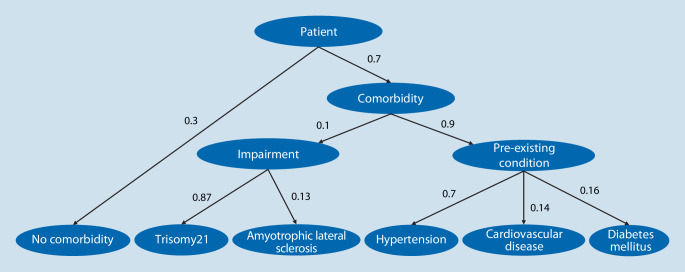
Probabilities for the assignment of impairments and pre-existing conditions based on (adjusted) prevalence rates

In the case of a patient with a comorbidity, the impairments and pre-existing conditions were assigned based on the (adjusted) prevalence rates ([17–23]; Fig. 3). It was assumed that a patient with a comorbidity had a 10% probability of having an impairment and a 90% probability of having a pre-existing condition. This ensured that all comorbidities considered were included in the real-world simulation.

Based on the assigned probability, a binary variable *y*_*i*_ was then assigned via a uniformly distributed random number in the interval [0,1]. The binary variable indicated whether a patient died in cases of intensive care treatment:

$$y_{i}=\begin{cases} 1 & if\,\textit{patient i dies in case of}\\&\textit{intensive care treatment}\,\\ 0 & else \end{cases}$$

This variable was assigned on the basis of the simulated probability of death.

In the case of initial ex ante triage based on survival probabilities, the patients with the highest survival or lowest mortality probabilities were now selected for intensive care treatment. After initial occupancy ($t=0$), at each of the consecutive points in time considered $t\in T=\{1{,}2{,}3\}$, a queue was generated and either random consecutive occupancy or ex post triage based on survival probabilities was performed. In random consecutive occupancy, 6 patients (10% of ICU capacity) each in the ICU and in the queue were randomly selected to release or receive ICU capacity. With consecutive ex post triage based on survival probabilities, ICU capacity was allocated according to the generated probabilities of death. For each of the points in time considered, a calculation of the prospective ICU mortality was performed for the cohort of patients treated at that point in time:

$$m_{t}=\frac{1}{B}\cdot \sum _{i\in I}y_{i}\ \forall t\in T$$

The calculation of mortality within each patient group, i.e., patients without impairments and pre-existing conditions (${m}_{t}^{n}$) and patients with impairments and pre-existing conditions (${m}_{t}^{v}$), was performed equivalently. In addition, the number of patients treated, discharged, and admitted in each patient group at each time point was calculated. Subsequently, the mean values as well as the standard deviations of all considered ratios were calculated. Analysis of variance (ANOVA) and post hoc tests were performed to validate the results.

To research the influence of the number of patients to be treated and thus evaluate the sensitivity, different combinations of the need at the time of initialization, $d\in D=\{70{,}90\}$, and the length of the queue, $w\in W=\{10{,}20{,}30{,}60\}$, were considered. In addition, to evaluate the impact of potential misestimation, the parameter $e\in E=\{0.9;1;1.1\}$ was introduced. The parameter indicated whether the estimate of the probability of death corresponded to the actual probability ($e=1$) or whether it was underestimated or overestimated by 10%. This resulted in the probability that was considered in the triage decision:

$$p_{i}=e\cdot p_{i}\ \forall e\in E, i\in I$$

**Fig. 4:**
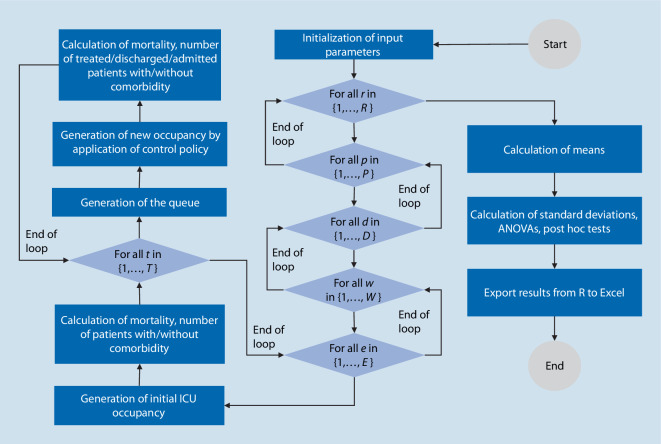
Flowchart of the simulation study in R (Statistical software, The R Foundation for Statistical Computing, Vienna, Austria)

An overview of the implementation in R is shown in Fig. 4.

## Results

**Fig. 5:**
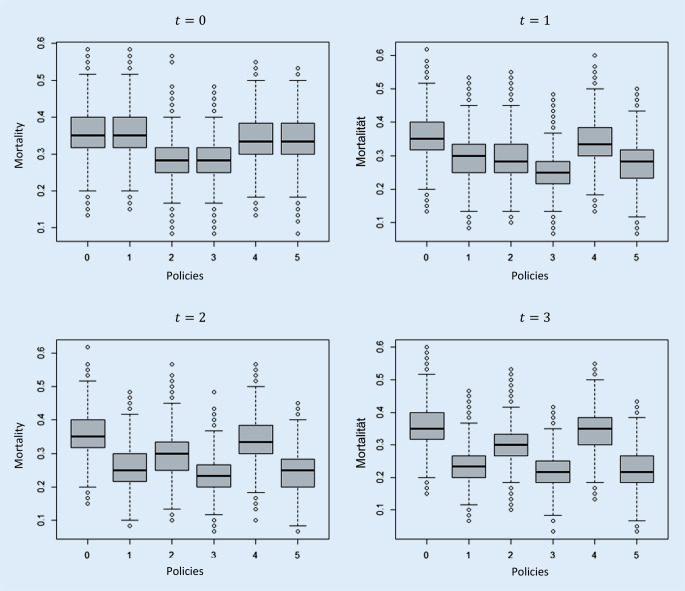
Boxplots of the mortality on ICU for a demand at the time of initialization of 70 patients, a queue of 10 patients and correct estimation of the mortality using different control policies. t = 0 initial situation and triage cycles t = 1–3

In this simulation study, mortality on the ICU is calculated in cases of different control policies and a situational overload of the system. In the following, the results for the real-world simulation scenario are presented with a need at the time of initialization of 70 patients (initial queue), a length of the queue of 10 (at time points 1, 2, and 3, respectively), and correct estimation of mortality, i.e., $e=1$. The average ICU mortality for each time point and control policy considered is shown in Fig. 5.

At the time of initialization there are no differences in mortality when policies 0 and 1, 2 and 3 as well as 4 and 5 are applied, because the initial occupancy is equivalent in these policies. Looking at consecutive points in time, the application of consecutive ex post triage based on survival probabilities leads to a reduction in (total) ICU mortality. On the first day ($t=1$) a reduction in total mortality from 33.81% to 27.87% is achieved for policy 5. This represents a relative reduction in mortality of approximately 18%. An overview of mortality in the individual patient groups per control policy for time $t=1$ (first ex post triage point in time) is shown in Table 3. It particularly shows the discrimination of the disabled by the present law: as compared to policy 5 (use of ex post triage), policy 4 (according to current legislature) produces 7.6% (35.3% vs. 42.9%) excess mortality in the disabled but only 0.1% in the patients without prior comorbidity or disability (15.6% vs. 15.7%). The results for the other points in time, the standard deviations and the number of patients treated per group can be found in the electronic appendix (supplementary material online: tables S1 and S2).

**Table 3:** Mortality per patient group and policy at time point t = 1 (real-world simulation). Table 2 provides descriptions of the policies

Policy	0 (%)	1 (%)	2 (%)	3 (%)	4 (%)	5 (%)
All patients	35.4	29.2	29.0	25.3	34.1	27.9
Patients without impairments and pre-existing conditions	15.9	15.7	16.1	16.0	15.7	15.6
Patients with impairments and pre-existing conditions	43.8	36.4	35.8	31.5	42.9	35.3

The evaluation of the variations and metrics examined lead to the following results: firstly, for the variations of need at the time of initialization (initial patients waiting for ICU treatment at time 0) and of the length of the queue (patients waiting for ICU treatment at time points 1, 2, and 3), triage leads to a higher reduction in mortality for an increasing number of patients to be treated. Secondly, varying parameter *e*, which indicates whether the probability of death of the patient was correctly estimated, underestimated or overestimated by 10%, has no significant effect on ICU mortality. This is because the probability of death is consistently underestimated/overestimated or correctly estimated in these scenarios. Thirdly, in the individual simulations in which a specific impairment or pre-existing condition is considered, the mortality-reducing effects of triage are shown to increase as the difference between the expected values of the patient groups increases. Fourthly, the results of ANOVA, which were performed to provide additional validation of the results, show statistically significant differences. The post hoc tests illustrate that means are significantly different in all pairwise comparisons. As expected, the initial occupancy is an exception because means are not significantly different between policies in which the same initial occupancy is performed.

## Discussion

The results show that the application of consecutive ex post triage based on survival probabilities leads to a reduction in both overall ICU mortality and mortality within individual ICU patient groups. In particular, the prediseased and disabled benefit significantly more from ex post triage than the patients without disabilities or comorbidities. Ex ante triage based on survival probabilities does lead to lower ICU mortality at the time of initialization, but mortality increases again in the case of random consecutive selection of patients. A combination of ex ante and ex post triage based on survival probabilities provides superior results, but pure ex ante triage is unlikely to ever be performed in practice because ICU occupancy is dynamic. Even ex ante triage for few free beds is unlikely, because patients arrive over time and decisions are made sequentially. Benefits in terms of ICU mortality can also be observed when focusing on different patient groups; however, patients with impairments and pre-existing conditions are disproportionately discharged or not admitted when consecutive ex post triage based on survival probabilities is performed. This is because the expected probability of death is bigger for this patient group than for patients without impairments and pre-existing conditions. Even though ex post triage based on survival probability leads to a reduction in ICU mortality in all patient groups, more patients with impairments and pre-existing conditions tend not to be treated (anymore); however, according to literature 70% of patients in the ICU are impaired or prediseased [16], and therefore a larger number of patients in this group will not be (further) treated compared to patients without impairments and pre-existing conditions. Moreover, the simulation results show that the mortality-reducing effects of triage are amplified when the number of patients to be treated, i.e., the need at the time of initialization and the length of the queue, increases. Finally, in this work, mortality reduction is considered an essential goal, even if discussions on accepting higher mortality in favor of random selection or other distribution procedures because of ethical reasons exist.

The study is subject to some limitations. First, it is assumed that all patients who cannot be treated immediately will die. Thus, the focus is exclusively on mortality on the ICU. Second, our model represents a situational overload of the system and excludes natural patient flows, i.e., the possibility of a transfer of patients due to an improvement of condition or death. Third, the survival probability of a patient is stable during the treatment on the ICU. In practice, however, the survival probability may change, possibly indicated by medical scores during the course of treatment, and thus management based on these scores would yield different results. Initially, ICU patients are treated under the assumption that intensive care treatment will save their lives; however, for many ICU patients, the probability of survival shifts considerably over time—both in one direction and the other. Selection of patients at consecutive time points is extraordinarily difficult unless a medical indication to continue treatment no longer exists or the patient’s will is against continuing ICU treatment. In addition, significantly longer treatment times resulted during the COVID-19 pandemic. Fourth, the parameters of the triangular distributions in the simulation are chosen in such a way that systematic discrimination of impaired and prediseased patients is excluded. This assumption is to be evaluated from a practical perspective. Fifth, the use of pure ex ante triage does not correspond to a realistic scenario, as an ICU occupancy grows organically during operation. In this case, the distribution of freed-up capacity is based on the patients’ need for intensive care treatment and not on the likelihood of success. Only in exceptional cases patients arrive at the same time and compete for ICU treatment as in ex ante triage. The idea of free ICU capacity and corresponding ex ante triage is unlikely. Sixth, based on characteristics of patients in German hospitals, the study assumes that 30% of ICU patients are not impaired or prediseased. In addition, the (adjusted) prevalence rates of various impairments and pre-existing conditions in Germany are applied. Consequently, a transfer of the results to other regions (different patient groups, deviating prevalence rates) is only possible to a limited extent.

## Conclusion

This study provides a simulation-based evaluation of different ex ante and ex post triage policies in the ICU under consideration of survival probabilities, impairments and pre-existing conditions. The results show that an implementation of consecutive ex post triage based on survival probabilities leads to a reduction in ICU mortality in all patient groups considered. Particularly serious, however, is the finding that the law significantly disadvantages disabled and prediseased people because it produces significantly more deaths among the disabled than among the non-disabled. Therefore, the exclusion of ex post triage is to be critically discussed in the light of the current legislation.

The aim of this work is to analytically research the effects of ex ante and ex post triage. For future research, the approach of data-driven decision support for the management of intensive care capacities during situational overload of the system may be pursued further. A proper data basis is of special importance. In addition, it is essential not to consider the results in isolation, but to discuss them in an interdisciplinary way from medical, practical, ethical and legal perspectives.

### Supplementary Information

Supplemental tables S1 and S2

## Footnotes

1 Minimum (0.04), maximum (0.41), and mode (0.045) of the probability of death.

2 Expectation

3 A density function describes the distribution of a continuous random variable. The area below the given density function describes the probability that the probability of death is at most a certain value. For example, the probability that the probability of death of patients with hypertension lies between 0.19 and 0.63 is 100%.

4 No evaluation of sequential ex-ante triage is performed. For (consecutive) ex-post triage, multiple points in time are considered.

5 The initial occupancy of the ICU occurs before the individual points in time are considered and is denoted as $t=0$. No ex-post triage is performed at initiation.

6 Combining the two initial forms of occupancy in Policies 4 and 5, a need of 12 patients was assumed for the beds to be occupied via ex-ante triage based on survival probabilities.

7 To validate the results, the probability of death was simulated 1000 times for each patient group and the expected values were calculated. These correspond to the calculated expected values of the triangular distributions when the proportionates were applied.

## Declarations

For this article no studies with human participants or animals were performed by any of the authors. All studies mentioned were in accordance with the ethical standards indicated in each case.

The supplement containing this article is not sponsored by industry.
