# Supplementary material for: Simulation of the mortality after different ex ante (secondary) and ex post (tertiary) triage methods in people with disabilities and pre-existing diseases
Source: Anaesthesiologie. 2023 Sep 21;72(Suppl 1):10–8. doi: 10.1007/s00101-023-01336-7 (PMC10692011; doi:10.1007/s00101-023-01336-7)
Supplement: Supplementary file 1 — Supplemental tables S1 and S2 [file 101_2023_1336_MOESM1_ESM.pdf]

Supplementary material to the article " Simulation of mortality after different ex-ante- (secondary) and ex-post- (tertiary) triage methods in people with disabilities and comorbidities" by Garber S, Brunner JO, Heller AR et al. (2023) in *Die Anaesthesiologie*.

The article and additional material are available at www.springermedizin.de. Please enter the title of the article in the search field.

Table S1: Mortality (mean and standard deviation) per patient group, triage policy and point in time (real-world simulation)

	t	Policy 0	Policy 1	Policy 2	Policy 3	Policy 4	Policy 5
All patients	0	0.354 (0.06)	0.354 (0.06)	0.283 (0.06)	0.282 (0.06)	0.339 (0.06)	0.338 (0.06)
Patients without impairments and pre-existing conditions	0	0.160 (0.08)	0.158 (0.08)	0.160 (0.08)	0.160 (0.08)	0.157 (0.08)	0.156 (0.08)
Patients with impairments and pre-existing conditions	0	0.437 (0.08)	0.437 (0.08)	0.348 (0.08)	0.348 (0.08)	0.428 (0.08)	0.427 (0.08)
All patients	1	0.354 (0.06)	0.292 (0.06)	0.290 (0.06)	0.253 (0.06)	0.341 (0.06)	0.279 (0.06)
Patients without impairments and pre-existing conditions	1	0.159 (0.09)	0.157 (0.08)	0.161 (0.08)	0.160 (0.08)	0.157 (0.08)	0.156 (0.08)
Patients with impairments and pre-existing conditions	1	0.438 (0.08)	0.364 (0.08)	0.358 (0.08)	0.315 (0.08)	0.429 (0.06)	0.353 (0.06)
All patients	2	0.354 (0.06)	0.256 (0.06)	0.296 (0.06)	0.233 (0.05)	0.342 (0.06)	0.245 (0.06)
Patients without impairments and pre-existing conditions	2	0.159 (0.09)	0.156 (0.07)	0.161 (0.08)	0.158 (0.07)	0.158 (0.08)	0.155 (0.07)
Patients with impairments and pre-existing conditions	2	0.438 (0.08)	0.322 (0.08)	0.366 (0.08)	0.294 (0.08)	0.430 (0.08)	0.312 (0.08)
All patients	3	0.355 (0.06)	0.234 (0.05)	0.302 (0.06)	0.219 (0.05)	0.344 (0.06)	0.225 (0.05)
Patients without impairments and pre-existing conditions	3	0.160 (0.09)	0.155 (0.07)	0.160 (0.08)	0.154 (0.07)	0.158 (0.08)	0.153 (0.07)
Patients with impairments and pre-existing conditions	3	0.438 (0.08)	0.297 (0.08)	0.374 (0.08)	0.280 (0.08)	0.431 (0.08)	0.289 (0.08)

Table S2: Average number of ICU patients per patient group, triage policy and point in time (real-world simulation)

	t	Policy 0	Policy 1	Policy 2	Policy 3	Policy 4	Policy 5
Patients without impairments and pre-existing conditions	0	18.0	18.0	21.0	21.0	19.7	19.7
Patients with impairments and pre-existing conditions	0	42.0	42.0	39.0	39.0	40.3	40.3
Patients without impairments and pre-existing conditions	1	18.0	20.9	20.7	23.9	19.6	22.6
Patients with impairments and pre-existing conditions	1	42.0	39.1	39.3	36.1	40.5	37.4
Patients without impairments and pre-existing conditions	2	18.0	23.8	20.4	26.6	19.4	25.6
Patients with impairments and pre-existing conditions	2	42.0	36.2	39.6	33.4	40.6	34.5
Patients without impairments and pre-existing conditions	3	18.0	26.6	20.2	29.0	19.2	28.3
Patients with impairments and pre-existing conditions	3	42.0	33.4	39.8	31.0	40.8	31.7
